# A Study on the Correlation Between Undergraduate Students’ Exercise Motivation, Exercise Self-Efficacy, and Exercise Behaviour Under the COVID-19 Epidemic Environment

**DOI:** 10.3389/fpsyg.2022.946896

**Published:** 2022-07-04

**Authors:** Fang Wang, Shiying Gao, Baoxia Chen, Chenyu Liu, Zhusheng Wu, Yan Zhou, Yan Sun

**Affiliations:** ^1^School of Physical Education, Sichuan University, Chengdu, China; ^2^Department of MA Filmmaking, University for the Creative Arts, Farnham, United Kingdom; ^3^Cyberspace Institute of Advanced Technology, Guangzhou University, Guangzhou, China

**Keywords:** exercise motivation, exercise self-efficacy, exercise behaviour, undergraduate students, COVID-19 epidemic

## Abstract

The outbreak of COVID-19 epidemic has influenced human beings from various aspects including physical exercise behaviours. This study aims to explore the influence of the COVID-19 epidemic on exercise self-efficacy and exercise behaviour, as well as the intermediary effects of exercise motivation. A sample of 1,115 undergraduate students was investigated using the physical exercise behaviour scale, exercise self-efficacy scale, and exercise motivation scale, combined with the COVID-19 epidemic environment as an influencing factor. SPSS was used for statistical analysis and AMOS for the prediction model building. Logical analysis was undertaken to sort out and analyse the data. The structural equation model reveals that exercise self-efficacy has a positive effect on exercise motivation and exercise behaviour. Meanwhile, the COVID-19 epidemic environment negatively influences exercise behaviour through the intermediary role of exercise self-efficacy and exercise motivation. Moreover, the intermediary effect of exercise self-efficacy is greater than that of exercise self-efficacy and exercise motivation. This study provides both theoretical implications and practical guidelines for society and undergraduate students to improve their exercise behaviour during epidemic.

## Introduction

The emergence and worldwide spread of the novel coronavirus epidemic (hereinafter referred to as the COVID-19 epidemic) has been deemed the most momentous event since the 21st century (WTTC, 2020). The COVID-19 epidemic surely brought about calamities with regard to many causalities for individuals’ socio-economic functioning and psychosocial states (Khudaykulov et al., 2022), which has influenced almost all the aspects of human beings, including sport and physical exercise. The long-term self-isolation resulting from the COVID-19 epidemic has challenged people to retain physical activity and quality of life (Dwyer et al., 2020). Because of the pandemic lockdowns in many areas worldwide, people’s exercise possibilities were limited to varying degrees, such as the closing of fitness centres (Constandt et al., 2020; Mutz and Gerke, 2020; Schnitzer et al., 2020). This limitation in physical exercise may lead to adverse outcomes because exercise contributes to disease prevention, chronic disease treatment, and maintenance of psychological well-being (Lavie et al., 2019; Rodríguez et al., 2020). It requires schools to make some changes regarding physical education. However, little research has been done on exactly how the COVID-19 environment has affected students’ exercise motivation and behaviour.

Research during COVID-19 has focused on physical health and exercise across various groups of people, including professional athletes (Schinke et al., 2020), children (Yarımkaya and Esentürk, 2020), and working adults (Zhang et al., 2020b). However, there is a dearth of studies on the effects of lockdown on undergraduate students, who tend to be the most energetic regarding physical exercise. Although the COVID-19 epidemic limited people’s outdoor exercises to some degree, young people’s exercise participation levels and patterns remain unclear. For example, evidence shows that young people in Germany are more likely to maintain participation in home-based sports and physical exercise during the COVID-19 epidemic, compared with older people who did not find adequate substitutes for their sporting routines (Mutz and Gerke, 2020). This indicates that it is still unclear how the quarantine affects physical exercise behaviour and efficacy. This study seeks to investigate the impact of the COVID-19 epidemic environment (EE) on undergraduate students’ exercise behaviour (EB), exercise self-efficacy (ES) and exercise motivation (EM). It considers China where the COVID-19 cases were first reported, as the research context, and Chinese undergraduate students whose daily life has been dramatically influenced by the epidemic, as representative.

The COVID-19 epidemic in China was severe in its early stage, and the government took various measures to prevent its spread. The closure of universities, sports facilities, and stadiums led to physical inactivity among Chinese undergraduate students. During the epidemic, 82.98% of Chinese undergraduate students exercised less than an hour a day (Hu et al., 2020). Recent research reveals that the exercise behaviour of Chinese undergraduate students during the COVID-19 epidemic has been affected by environmental, social, and psychological factors (Zhang et al., 2020a). However, existing research has failed to demonstrate this by explaining the mechanisms of that influence. This study explores the effects of the epidemic on the physical exercise behaviour of undergraduate students by constructing a structural equation model through multiple linear regression analysis. Implications are considered for undergraduate students and universities’ physical education to maintain health during and after the COVID-19 pandemic.

## Literature Review

### Exercise Self-Efficacy, Exercise Motivation, and Exercise Behaviour

Self-efficacy is a concept that originated from the social cognitive theory (SCT), which posits that changes in behaviour are accomplished through an intermediary cognitive mechanism (Bandura, 1977). Bandura (1986) proposed the theory of self-efficacy, stating that individual behaviour, cognition, and environment influence each other. Positive attitude towards one’s self-efficacy is a resource that may be utilised as a coping strategy in reducing stressors (Godinic et al., 2020). Being applied to physical exercise (Ryan et al., 1997), self-efficacy refers to the consideration of people’s ability to smoothly proceed or adhere to physical exercise behaviour in various environments. Exercise self-efficacy is the degree of confidence individuals have in their regular exercise. Studies have proved that self-efficacy is a significant predictor of physical activity (Dzewaltowski, 1989; Yordy and Lent, 1993; Armitage and Conner, 1999). It has a significant impact on the frequency of physical exercise behaviour (McAuley et al., 2000). In addition, self-efficacy has been found to significantly correlate with the total time spent on exercising (Lim et al., 2005), which is indispensable for the emergence and persistence of physical exercise behaviour. Conversely, exercise self-efficacy is related to a positive emotional response (McAuley et al., 2003), indirectly affecting exercise behaviour by increasing exercise motivation (Kwan and Bryan, 2010).

Bandura (1977) believes that motivation can dominate and guide behaviour to a certain extent. Different motivations may determine people’s participation in exercise due to the influence of gender, age, culture, level of experience, and type of skills (Brodkin and Weiss, 1990). Deci and Ryan (1985) put forward the self-determination theory, which divides motivation into three categories: internal motivation (an innate tendency of human beings to pursue novelty and challenge, develop and exercise their abilities, and dare to explore and learn), external motivation (people are not interested in the activity itself, but tend to engage in an activity to obtain some separable result), and no motivation (individuals do not make a connection between their behaviour and its results, and have no interest in the activity they are engaged in). Self-determination theory has been used to explain human exercise behaviour. Internal motivation can predict exercise behaviour, and provide strong evidence for long-term exercise (Ardeńska et al., 2016). In addition, a lack of internal motivation or external motivation is closely related to an avoidance of exercise behaviour (Standage et al., 2012).

Physical exercise behaviour is affected by various factors, including demographic, economic, and social factors (Becker and Maiman, 1975). Protection motivation theorists highlight that people’s participation in sports and exercise is often related to health-focused motivation (Rogers, 1983). People with knowledge and inspiration pertaining to health tend to have a stronger motivation for physical exercise (Becker and Maiman, 1975). Lim (2009) demonstrates that exercise behaviour is linked to exercise motivation and self-efficacy. The degree of self-efficacy will affect on the relationship between exercise motivation and behaviour (Klompstra et al., 2018). When self-efficacy is low, a higher degree of motivation will not promote exercise behaviour. The combination of higher self-efficacy and higher motivation will lead to exercise behaviour. Furthermore, exercise behaviour is also influenced by the interaction of multiple factors: society, family, school, unit, and other environments (Spence and Lee, 2003).

To summarise, the factors that affect individuals’ participation in physical exercise are not single but diverse. The antecedents that affect physical exercise behaviour mainly include psychological, individual, social, and environmental factors. The lack of sports venues and equipment and changing weather conditions are all factors that may affect people’s participation in physical exercise. The outbreak of COVID-19 has undoubtedly altered the physical exercise environment. Therefore, this study integrates the environmental factor of COVID-19 to explore the relationships among physical exercise behaviour, exercise motivation, and exercise self-efficacy.

### The Theory of Planned Behaviour

Ajzen (1991) developed the theory of planned behaviour (TPB), proposing that three independent determinants (attitude towards behaviour, subjective norms, and perceived behaviour control) are effective in the prediction of behavioural intention. The TPB (Ajzen, 1991) has been widely used in predicting and explaining human behaviour, such as customer behaviour (Liao et al., 2007), smoking behaviour (Karimy et al., 2015), exercise behaviour (Blue, 2010) and so on. Attitude towards behaviour is considered the most reliable variable in explaining behavioural intention (Ajzen, 1991). Subjective norms are social factors that refer to the perceived social pressure on implementing the behaviour. This indicates that an individual’s behaviour is influenced by the society they are part of. Subjective norms indirectly affect behaviour through their influence on behavioural intention (Ajzen, 1991). Compared to the other variables, subjective norms have the least influence on behavioural intention (Terry et al., 1999).

Perceived behaviour control (PBC) refers to the perceived difficulty of executive behaviour and an individual’s judgement of their ability to control specific behaviours (Ajzen, 1991). By applying PBC in different contexts, including exercise (Hagger et al., 2002), leisure (Ajzen and Driver, 1992), and health (Albarracin et al., 2001), its usefulness in predicting behaviour has been verified. The more positive the attitude and subjective norms, the greater the PBC, and the stronger the behavioural intention. Behavioural intention is the most direct factor affecting behaviour. However, in practice, behaviours may encounter many difficulties in the process of implementation, which may, in turn, restrict the intention of the behaviour. There is a reciprocal relationship between behaviour and behavioural intention.

### Research Framework and Hypotheses

This study integrates the TPB (Ajzen, 1991) and the SCT (Bandura, 1977) to predict physical exercise behaviour. The behavioural intention from the TPB and the self-efficacy from the SCT are the effective factors used to predict exercise behaviour. According to the TPB, subjective norms, sense of control, and attitude towards behaviour can be adapted to explain and predict exercise behaviour. In the field of exercise behaviour, behavioural intention is replaced by exercise motivation. It considers not only the psychological motivation for people to retain exercise behaviour but also the direct reasons and motivations for the exercise behaviour. In the field of exercise behaviour, exercise self-efficacy refers to the confidence required of individuals to perform continuous and regular exercise under various conditions and to regulate individuals’ estimation of their cognition of participating in exercise behaviour.

**Figure 1:**
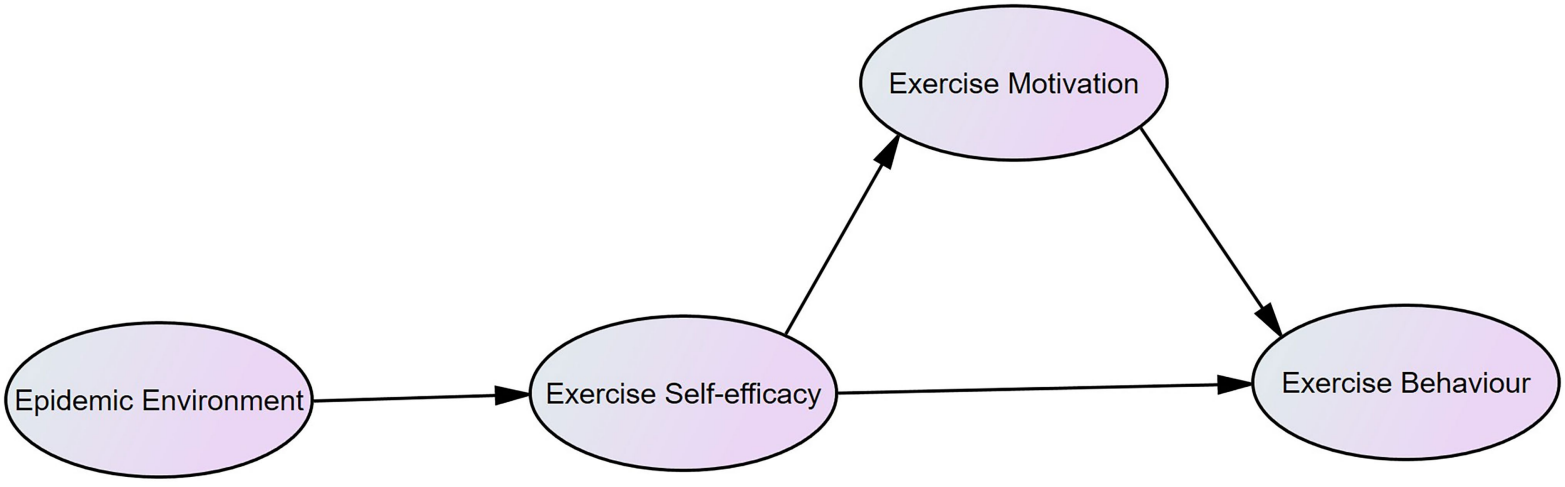
The path map of the influence of the COVID-19 epidemic environment on physical exercise motivation, self-efficacy, and behaviour.

With regard to the influence of environment on physical exercise behaviour, the COVID-19 epidemic environment was integrated into the theoretical framework of this study (Figure 1). The undergraduate students’ physical exercise behaviour (EB) was considered as the explained variable, and the COVID-19 epidemic environment (EE), physical exercise motivation (EM), and exercise self-efficacy (ES) were considered as explanatory variables. This study assumes that the COVID-19 epidemic environment can indirectly affect physical exercise behaviour, through the factors of physical exercise motivation and exercise self-efficacy. The structural equation model (SEM) was used to verify whether the theoretical framework path was reasonable and explore the influencing factors of physical exercise behaviour.

People experience difficulties in maintaining physical activity and quality of life due to the COVID-19 epidemic (Dwyer et al., 2020). Most people engage less in sports and physical exercise under quarantine (Schnitzer et al., 2020), including Chinese undergraduate students (Hu et al., 2020; Zhang et al., 2020a). Therefore, we propose hypothesis one (H1): the COVID-19 epidemic environment is negatively correlated with exercise self-efficacy, which in turn affects undergraduate students’ participation in physical exercise. Exercise self-efficacy has been confirmed to affect the choice of exercise behaviour, the persistence and effort involved in the exercise behaviour, and further, the individual’s exercise motivation level, attribution of success or failure, performance results, and emotional response (McAuley et al., 2000, 2003; Kwan and Bryan, 2010). Similarly, exercise behaviour is positively affected by self-efficacy, while exercise self-efficacy has a positive effect on motivation (Lim, 2009). According to this, hypotheses two (H2) and three (H3) are proposed as follows: H2 posits that exercise self-efficacy is positively correlated with exercise motivation, promoting undergraduate students’ participation in physical exercise, and H3 posits that exercise self-efficacy is positively correlated with physical exercise behaviour, stimulating undergraduate students’ participation in physical exercise. Previous studies have verified the positive influence of motivation on physical exercise participation (Duncan et al., 2010; Standage et al., 2012). Thus, we propose hypothesis four (H4): Physical exercise motivation is positively correlated with physical exercise behaviour, promoting undergraduate students’ participation in physical exercise.

## Materials and Methods

### Participants

The sample of this study were participants of 1,300 undergraduate students in China, geographically including students from east, west, north, south, and central China. And the proportion of male and female in the survey sample were relatively balanced: the percentage of male and female is 47.7% and 52.3% accordingly.

### Evaluation Instrument Design

The evaluation instrument in this study was a sum of questionnaires designed based on the exercise self-efficacy scale (Benisovich et al., 1998; Li, 2010), physical exercise motivation scale (Ryan et al., 1997; Chen et al., 2013), and physical exercise behaviour scale (Li, 2018). In addition, questions about the influence of the COVID-19 epidemic environment were added. To evaluate each scale, a seven-point Likert scoring method was used (e.g., 1: strongly agree, 7: strongly agree).

As for the explained variable, physical exercise behaviour, the undergraduate students’ physical exercise behaviour scale (Li, 2018) developed by Li (2018) was adopted. According to Li (2018), the scale included seven dimensions: autonomous exercise behaviour (i.e., you always exercise consciously), exercise promotion behaviour (i.e., you will ask professionals to guide your physical exercise), exercise planning behaviour (i.e., you will make detailed exercise plans), situation-induced behaviour (i.e., you will exercise hard in order to participate in sports competitions), goal outcome behaviour (i.e., as long as you feel tired, it is hard for you to keep exercising), passive participation behaviour (i.e., for some reasons, such as illness or obesity, you are forced to keep exercising), and injury coping behaviour (i.e., after injury, you will take the initiative to seek help from experienced people). Among seven dimensions, six of the 20 topics were selected according to the research objectives. Situation-induced behaviour was deleted because it was not related to our study. In considering the explained variable, the physical exercise behaviour of undergraduate students had a certain exercise intensity and time. It consisted of performing physical activities in a physical education class and in their spare time keeping fit and maintain a good physical condition.

Regarding the explanatory variable, the COVID-19 epidemic environment, we designed a specific scale based on three dimensions. Considering that students’ exercise methods and exercise environment were changed during the epidemic, and both of their physical state and psychological state were affected. Thus, the original scale included three dimensions of physical factors, psychological factors and external environment factors. The items were generated by analysing the potential impact of the epidemic environment on exercise through a board literature review. The scale was then evaluated and revised by experts followed by field testing in our sample. Factor analysis were applied to examine the scale’s construct validity and reliability. The dimension of psychological factors was deleted in factor analysis. The final scale consists of two dimensions with seven items (Appendix). The dimension of physical factors includes item such as “due to the COVID-19 epidemic environment, you have to stay at home for a long time and feel listless,” and the dimension of external environment factors includes item such as “due to the COVID-19 epidemic environment, there is no place to exercise because of the closure of fitness centres and sports facilities.” Therefore, a higher score means a greater extent that the individual was influenced by the COVID-19 epidemic environment.

The exercise self-efficacy scale (Benisovich et al., 1998) revised by Li (2010) was adopted to test the explanatory variable of exercise self-efficacy. The scale included four dimensions: physical (i.e., when you feel tired, you can keep exercising), activity (i.e., when you did not achieve your exercise goal, you can keep exercising), mental (i.e., when you feel depressed, you can keep exercising), and conflict factors (i.e., when you have other appointments, you can keep exercising). Nine topics were selected to evaluate participants’ ability and confidence in their regular, continuing exercise behaviour. The exercise motivation scale (Ryan et al., 1997) revised by Chen et al. (2013) was adopted to test the explanatory variable of exercise motivation. This scale included the following dimensions: social motivation (i.e., you keep exercising because you want to make new friends), appearance motivation (i.e., you keep exercising because you want to be more attractive to others), ability motivation (i.e., you keep exercising because you want to improve your sports skill), health motivation (i.e., you keep exercising because you want to keep healthy), and enjoyment motivation (i.e., you keep exercising because sports are entertaining). Furthermore, two further dimensions (institutional and obedience motivation; i.e., you keep exercising in order to coping with the physical exercise examination) were added to create a total of seven dimensions, which should measure participants’ external motivations according to the self-determination theory (Deci and Ryan, 1985). Nine out of 37 topics were selected on the basis of their relevance.

### Data Collection

Given the quarantine and lockdown measures of the COVID-19 pandemic, an electronic version of the questionnaire was posted on the website “Questionnaire Star”,^1^ which was open for each visitor to access. The initial contact with the potential participants was taken as we sent the questionnaires *via* the most popular social media application for Chinese youth, WeChat. Specifically, we distributed the questionnaire link to 27 WeChat groups for undergraduate students between August 20 and 28, 2020. A total of 1,300 questionnaires were distributed to students and completed by them. Among the 1,300 questionnaires, 1,115 are valid, with an effective rate of 85.77%. The criteria for excluding unqualified samples were incomplete answers, same answer for every question, and less than 90 s (“Questionnaire Star” measures the time they took to answer the questions). The research model used was a typical structural equation model.

### Statistical Analysis

In this study, SPSS 22.0 (International Business Machines Corporation, New York) was used to analyse the preliminary reliability and validity of the data and exploratory factor analysis. AMOS 23.0 (International Business Machines Corporation, New York) was used to construct the structural equation model for detailed data analysis, which was modified to achieve better fitting and adaptability. The data were then analysed to explore the relationships of COVID-19 epidemic environment, undergraduate students’ exercise behaviour, exercise self-efficacy and exercise motivation.

## Results

### Descriptive Statistics

In the analysis of the sample structure, the sex ratio was 47.7% male 52.3% female, which was reasonable. As for the students’ geographical location, the participants were distributed across all 32 provincial administrative regions in China.

### Reliability Test

In this study, confirmatory factor analysis (CFA) was used to verify constructive validity. When the data show good constructive validity, they must have convergent validity and discriminant validity.

SPSS 22.0 was used to test the reliability and validity of the questionnaire. As shown in Table 1, the Cronbach’s Alpha for each scale was greater than 0.8, and the Kaiser–Meyer–Olkin (KMO) greater than 0.8. Each value conforms to the reliability and validity requirements of the questionnaire.

**Table 1:** Reliability test of the questionnaire.

Item	Cronbach’s alpha	KMO	Questions (*n*)
Full questionnaire	0.905	0.953	31
EE	0.869	0.875	7
EM	0.956	0.949	9
ES	0.933	0.936	9
EB	0.911	0.880	6

*EE, Epidemic environment; EM, Exercise motivation; ES, Exercise self-efficacy; EB, Exercise behaviour; KMO, The Kaiser–Meyer–Olkin*.

### Validity Test

A convergent validity test with average variance extracted (AVE) was used to test the convergent and discriminant validity. Table 2 shows that the AVE of each dimension is greater than 0.5, proving that the convergence degree of the model is good. The correlation coefficients of exercise behaviour, exercise motivation, exercise self-efficacy, and the COVID-19 epidemic environment are less than 0.7. Only one item is greater than 0.7, but the excess coefficient is not high; within the permitted range, it is acceptable. The square roots of the diagonal AVE values in the table (indicated in bold) are larger than the correlation coefficients between the dimensions shown below the diagonal values. Therefore, there is a significant difference in validity among the dimensions.

**Table 2:** Validity test of the questionnaire.

	Convergent validity	Pearson correlation and discriminant validity			
	AVE	EE	ES	EM	EB
EE	0.560	**0.748**			
ES	0.609	−0.341	**0.780**		
EM	0.688	−0.192	0.563	**0.829**	
EB	0.602	−0.262	0.768	0.642	**0.776**

*Boldface numbers are square-root AVEs. EE, Epidemic environment; ES, Exercise self-efficacy; EM, Exercise motivation; EB, Exercise behaviour; AVE, Average variance extracted*.

### Fitness Test of the Measurement Model

The main purpose of the model fitness test is to verify the degree of the fit between the theoretical model and the actual data of the structural equation model. The better the goodness of fit of the model, the better the match between the theoretical model and the actual data. A series of fit indices were tested, including the goodness-of-fit statistic (GFI), the adjusted goodness-of-fit statistic (AGFI), root mean square error of approximation (RMSEA), relative/normed chi-square statistic (CMIN/DF), and the standardised root mean square residual (SRMR; Hooper et al., 2008). As shown in Table 3, all fitness indexes in the model are in line with the standard, indicating that the fitness of this model is good.

**Table 3:** Index table of model fitness.

Model fitting index	Standard	Model fitting degree	Model fitting judgement
GFI	>0.9	0.944	Modification
AGFI	>0.9	0.929	Modification
RMSEA	<0.08	0.051	Modification
CMIN/DF	<5	3.907	Modification
SRMR	<0.08	0.045	Modification

*GFI, Goodness-of-fit index; AGFI, Adjusted goodness-of-fit index; RMSEA, Root mean square error of approximation; CMIN/DF, Discrepancy divided by degree of freedom; SRMR, Standardised root mean squared residual*.

### Model Analysis of the Linear Structural Equation

The structure of the model integrating COVID-19 epidemic environment factor is significant for parameter estimation (Table 4), that is, *p* < 0.001. Under the standardisation of item reliability, the factor load (EE6 = 0.718, EE5 = 0.786, EE4 = 0.823, EE1 = 0.655), is greater than 0.6 and the SMC value is greater than 0.36. These are within the acceptable range, proving that the topic of the COVID-19 epidemic environment scale shows good validity. When (CR = 0.835) is greater than 0.8, it proves that the structure has good internal consistency. The convergent validity (AVE = 0.560) is greater than 0.5, within the acceptable range, indicating that the model and its convergent validity are good. The measurement model for exercise self-efficacy is significant in parameter estimation, that is, *p* < 0.001.

**Table 4:** Confirmatory factor analysis.

		Parameter significance estimation				Topic reliability		Composition reliability	Convergent validity
		Unstd.	S.E.	*Z*-value	Value of *p*	Std.	SMC	CR	AVE
EE6	EE	1				0.718	0.515	0.835	0.560
EE5	EE	1.165	0.05	23.15	***	0.786	0.618		
EE4	EE	1.173	0.049	23.793	***	0.823	0.677		
EE1	EE	0.916	0.046	19.735	***	0.655	0.429		
ES9	ES	1				0.755	0.569	0.903	0.609
ES8	ES	0.969	0.035	27.869	***	0.811	0.658		
ES4	ES	1.037	0.035	29.356	***	0.85	0.722		
ES3	ES	0.946	0.039	24.193	***	0.715	0.512		
ES2	ES	0.992	0.039	25.624	***	0.753	0.567		
ES1	ES	0.959	0.035	27.133	***	0.792	0.628		
EM2	EM	1				0.826	0.681	0.930	0.688
EM3	EM	1.033	0.028	36.972	***	0.887	0.787		
EM4	EM	0.956	0.032	30.284	***	0.778	0.606		
EM5	EM	0.945	0.032	29.586	***	0.766	0.587		
EM7	EM	0.919	0.027	33.551	***	0.834	0.696		
EM9	EM	1.034	0.028	36.41	***	0.879	0.772		
EB2	EB	1				0.843	0.710	0.858	0.602
EB3	EB	0.887	0.032	27.749	***	0.749	0.561		
EB4	EB	0.905	0.037	24.659	***	0.685	0.469		
EB5	EB	1.042	0.033	31.242	***	0.818	0.670		

*** *A statistical significance of *p* < 0.001*.

*Unstd., Unstandardised Estimate; S.E., Standard error; Std., Standardised estimate; SMC, Squared multiple correlations; CR, Component reliability; AVE, Average variance extracted value; EE, Epidemic environment; ES, Exercise self-efficacy; EM, Exercise motivation; EB, Exercise behaviour*.

Under the standardisation of item reliability, the factor load (ES9 = 0.755, ES8 = 0.811, ES4 = 0.85, ES3 = 0.715, ES2 = 0.753, ES1 = 0.792) is greater than 0.6, and the SMC value is greater than 0.36. These are well within the acceptable range, proving that exercise self-efficacy has good validity. The composition reliability (CR = 0.903) is greater than 0.8, which proves that the structure has good internal consistency, and the convergent validity (AVE = 0.609) is greater than 0.5, indicating that the model and its convergent validity are good. Both the questions and the structure of the exercise motivation measurement model are significant in parameter estimation, that is, *p* < 0.001. Under the standardisation of item reliability, the factor load (EM2 = 0.826, EM3 = 0.887, EM4 = 0.778, EM5 = 0.766, EM7 = 0.834, EM9 = 0.879) is greater than 0.7, and the SMC value for each question is greater than 0.5. These are in a good range, proving that the exercise motivation topic itself has good validity. The composition reliability (CR = 0.930) is greater than 0.8, which proves that the structure itself has good internal consistency, and the convergent validity (AVE = 0.688) is greater than 0.5, in a good range, indicating that the model and its convergent validity are good.

Both the questions and the structure of the exercise behaviour measurement model are significant in parameter estimation, that is, *p* < 0.001. Under the standardisation of the item reliability, the factor load (EB2 = 0.843, EB3 = 0.749, EB4 = 0.685, EB5 = 0.818) is greater than 0.6, and the SMC value is greater than 0.36, which is within the acceptable range, proving that the exercise behaviour problem itself has good validity. When CR = 0.858, is greater than 0.8, it proves that the structure itself has good internal consistency, and the convergent validity (AVE = 0.602) is greater than 0.5, in the acceptable range, indicating that the model and its convergent validity are good.

### Verification and Analysis of Research Hypotheses

In consideration of hypothesis testing and analysis (see Table 5), the COVID-19 epidemic environment has a negative effect on exercise self-efficacy (β = −0.341); exercise self-efficacy has a positive impact on exercise behaviour (β = 0.563 and 16.899, *p* < 0.001). Exercise self-efficacy has a positive effect on exercise motivation (β = 0.595), and exercise motivation has a positive effect on exercise behaviour (β = 0.308). Therefore, we can assume that H1, H2, H3, and H4 are all verified.

**Table 5:** Analysis of the research hypotheses.

Path relationship	Standardisation coefficient	*T*-value	Value of *p*	Supported
H1: EE-ES	−0.341	−9.675	***	Yes
H2: ES-EM	0.563	16.899	***	Yes
H3: ES-EB	0.595	17.338	***	Yes
H4: EM-EB	0.308	10.380	***	Yes

*** *A statistical significance of *p* < 0.001*.

*EE, Epidemic environment; ES, Exercise self-efficacy; EM, Exercise motivation; EB, Exercise behaviour*.

### Analysis of the Intermediary Effect of the Model

**Figure 2:**
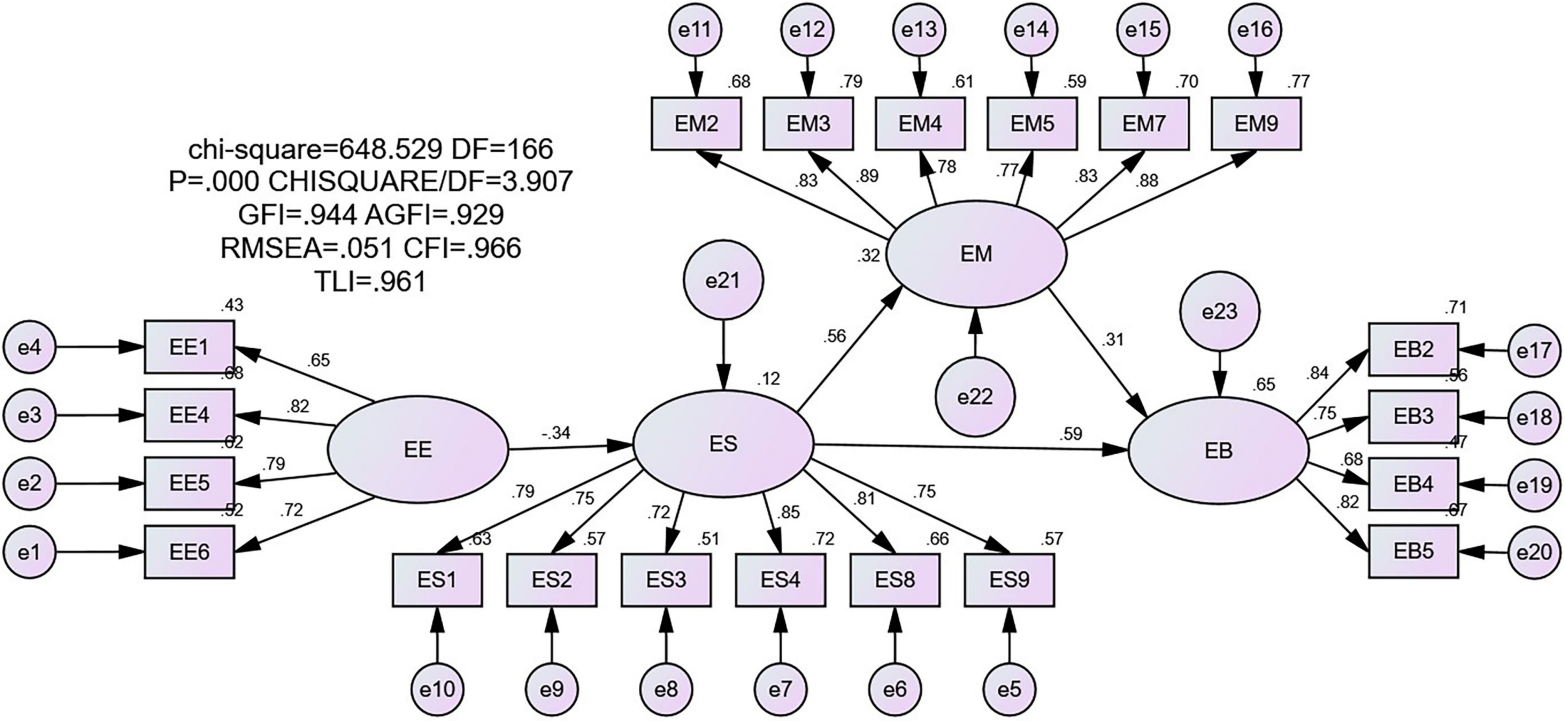
Research framework model.

In this study, Amos’s structural equation model was used to analyse the intermediary role of exercise self-efficacy and exercise motivation on exercise behaviour in the COVID-19 epidemic environment. Figure 2 shows the model fitting index (RMSEA = 0.051, GFI = 0.944, AGFI = 0.929, SRMR = 0.0451), showing that the model fits well. The COVID-19 epidemic environment has a negative effect on exercise self-efficacy (β = −0.341), while exercise self-efficacy has a positive effect on exercise behaviour (β = 0.563). Therefore, exercise self-efficacy plays an intermediary role between exercise behaviour and the COVID-19 epidemic environment. Exercise self-efficacy has a positive effect on exercise motivation (β = 0.595), while exercise motivation has a positive effect on exercise behaviour (β = 0.308). Exercise self-efficacy and exercise motivation play a remote intermediary role in exercise behaviour in the COVID-19 epidemic environment.

In this study, the Bootstrap method was used to test the intermediary effect by re-sampling 5,000 times. As shown in Table 6, the intermediary effect of exercise self-efficacy is 0.187, accounting for 77.6% of the total intermediary effect. The lower limit and upper limit values of 95% CI and percentile 95% CI do not include the value 0. Therefore the indirect effect is established, indicating that there is an intermediary effect between the COVID-19 epidemic environment and exercise behaviour in this study. The effect value of the remote intermediary effect of exercise self-efficacy and exercise motivation is 0.054, accounting for 22.4% of the total intermediary effect. The lower and upper limits of CI and percentile 95% CI do not include 0. Therefore, the indirect effect is established. This shows an intermediary effect between exercise self-efficacy and exercise motivation in this study between the COVID-19 epidemic environment and exercise behaviour. According to the comparison of the indirect effect and the proportion of the intermediary effect, the intermediary effect of exercise self-efficacy is greater than that of exercise self-efficacy and exercise motivation.

**Table 6:** Analysis of the intermediary effects of the model.

	Point estimate	Product of coefficient		Bootstrap 1,000 times 95% CI			
				Bias-corrected		Percentile	
		SE	Z	Lower	Upper	Lower	Upper
EE-ES-EB	0.187	0.028	6.68	0.135	0.243	0.135	0.243
EE-ES-EM-EB	0.054	0.010	5.4	0.038	0.077	0.036	0.075
Total indirect effects	0.241	0.032	7.53	0.179	0.304	0.179	0.304
Contrasts							
ES vs. ES-EM	0.132	0.027	4.89	0.085	0.189	0.084	0.188

*EE, Epidemic environment; ES, Exercise self-efficacy; EM, Exercise motivation; EB, Exercise behaviour*.

## Discussion and Conclusion

The results of this study suggest that the epidemic situation of COVID-19 affects undergraduate students’ participation in physical. There is a positive mutual correlation between undergraduate students’ exercise motivation, exercise self-efficacy, and exercise behaviour, similar to the findings of Standage et al. (2012) and Ardeńska et al. (2016). Through model analysis, H1 (β = −0.341) was accepted, indicates that the COVID-19 epidemic environment has a negative effect on exercise self-efficacy; H2 (β = 0.563) was accepted, indicates that exercise self-efficacy has a positive effect on exercise behaviour; H3 (β = −0.595) was accepted, indicates that exercise self-efficacy has a positive effect on exercise motivation; H4 (β = 0.308) was accepted, indicates that exercise motivation has a positive effect on exercise behaviour. The reduction in participation in physical exercise during the COVID-19 epidemic is traceable in “laziness and fatigue,” “lack of motivation,” “lack of appropriate facilities/equipment/space” (Farah et al., 2021). This is consistent with the results of the study. According to questionnaires, some undergraduate students experienced physical laziness as they reported tiredness and therefore decreased physical activity, while some students decreased physical activity due to lacking motivation. Besides, the closure of exercise venues leads to a reduction in opportunities to exercise (Constandt et al., 2020; Schnitzer et al., 2020) making it more difficult for students to participate in physical exercise.

The remote effect of the COVID-19 epidemic environment on exercise behaviour are novel. To explore the effect of epidemic on students’ exercise behaviour, a new scale of the epidemic environment was developed. The COVID-19 situation does not directly affect exercise behaviour, but rather that effect takes place through the mediation of exercise self-efficacy and exercise motivation. The negative influence of the COVID-19 epidemic environment on undergraduate students’ self-efficacy results in a decline in their exercise motivation, which further leads to a decline in exercise behaviour. The psychological changes of the COVID-19 epidemic environment result in the main effects on exercise behaviour. Previous studies showed that the COVID-19 pandemic has impacted mental health and created significant challenges for society (Maugeri et al., 2020; Guberina and Wang, 2021; Khudaykulov et al., 2022). As for undergraduate students, empirical studies have reported the prevalence of anxiety and stress during the COVID-19 epidemic (Chang et al., 2020; Liu et al., 2020; Saddik et al., 2020; Lee et al., 2021). This can be attributed to the fear of disease, such as being infected when going out for exercise (Diamond and Waite, 2020). It has been shown that exercising during COVID-19 increases the risk of infection (Woods et al., 2020). According to Maslow’s demand theory (Maslow, 1943), in the face of the COVID-19 pandemic, people must first ensure that their lives are not at risk before considering social or self-fulfilment needs. Therefore, exercise self-efficacy and exercise opportunities of undergraduate students in the COVID-19 epidemic environment are reduced due to the students’ concerns over their own safety, resulting in the decrease in exercise behaviour. The COVID-19 pandemic has forced Chinese undergraduate students to stay at home, leading to a reduction in their physical activity, and an increase in sedentary behaviour (Yeo, 2020). The feeling of fear, anxiety, and stress due to home confinement further impacted their motivation to continue exercises (Kaur et al., 2020).

This study has some practical applications that are worth highlighting. The findings of the study indicate that the negative effect of COVID-19 environment on students’ exercise behaviour takes place through the mediation of exercise self-efficacy and exercise motivation. Thus, more importance should be attached to promoting undergraduate students’ exercise self-efficacy and exercise motivation. Exercise self-efficacy is an essential intermediary factor between exercise behaviour and the COVID-19 epidemic environment. Faced with stress caused by the COVID-19 epidemic, gaining self-efficacy could be helpful in reducing stressors and promoting mental health (Godinic et al., 2020). To enhance students’ exercise self-efficacy, we need to consider measures that parents, schools, and society can take to maintain students’ mental health during the pandemic. We should also advocate psychological research on students to avoid students’ psychological anxiety, depression, and negative emotions due to the impact of the pandemic. This is particularly important in the COVID-19 pandemic situation because the physical education class is losing its identity as a consequence of online touchless classes (Varea and González-Calvo, 2020).

In addition, information about different possibilities of home exercise programs should be provided (Schwendinger and Pocecco, 2020). Many types of sports and exercise can be performed at home, such as yoga and meditation (National Center for Complementary and Integrative Health, 2020), rope skipping and jogging in place (Schwendinger and Pocecco, 2020). Undergraduate students should choose their own feasible and home-based physical activities to be performed daily to improve their immunity and prevent the novel coronavirus infection. To increase students’ exercise motivation, autonomy-supportive intervention in physical education should be provided since it is proved to be effective in enhancing autonomous motivation and physical activities in leisure-time (Barkoukis et al., 2020). Some incentive measures could be added, such as examining undergraduate students’ exercise situation at home through online methods and rewarding students who insist on exercising when returning to universities. Besides, making physical activity a regular part of their daily or weekly schedule and writing it on the calendar or exercising with relatives during quarantine can motivate themselves to exercise (Farah et al., 2021). Previous studies indicated that people were motivated when they observed others doing fitness activities (Kaur et al., 2020). During the quarantine, using social media, such as fitness apps, could be helpful for undergraduate students as they can be connected to each other and witness others’ activities. The empirical evidence also proved a higher use of social media and app use for home-based fitness activities (Tate et al., 2015; Ammar et al., 2020; Bagherian et al., 2021).

Furthermore, the findings of this study could be extended to the common public to help them gain exercise self-efficacy and exercise motivation and encourage them to participate in physical exercises, which would be beneficial to both their physical health and psychological health. There are some limitations to this study, which suggest directions for future research. Firstly, large-scale public health events such as the COVID-19 pandemic are infrequent, and the effects of such events vary. This indicates a challenge for generalisations made based on this study. Secondly, there are many factors that affect undergraduate students’ exercise behaviour. Exercise self-efficacy and exercise motivation are only a part of them. This research model can be extended upon in future research, and the factors that affect undergraduate students’ physical exercise behaviour can be analysed by analogy. Thirdly, it has to be considered that demographic factors, such as gender, age, and regional location or origin, may affect the relationship between exercise motivation and behaviour. Even for university students, the age span is relatively large, and exercise behaviour of newly enrolled and graduating students may be different. Therefore, future research could further investigate these factors. As a fourth point, attention needs to be paid to the geographical factors as this study considers China as the research context, because national pandemic strategy in China is quite different from those of many other countries, and the epidemic prevention policies of different provinces in China are also different. Thus, overlooking the political, social, and cultural differences that would apply to studying different regions. Future research may further explore the situation in countries suffering from COVID-19 following on China.

## Data Availability Statement

The raw data supporting the conclusions of this article will be made available by the authors, without undue reservation.

## Ethics Statement

Ethical review and approval was not required for the study on human participants in accordance with the local legislation and institutional requirements. Written informed consent from the (patients/participants or patients/participants legal guardian/next of kin) was not required to participate in this study in accordance with the national legislation and the institutional requirements.

## Author Contributions

FW, SG, BC, and CL formed the conception of the study and drafted the manuscript. YZ and ZW collected and analysed the data. YS contributed to the conception, design, and revision of the study. All authors contributed to the article and approved the submitted version.

## Funding

This paper was supported by the National Social Science Fund Projects “The research on intelligent elderly care service mode of sports and medicine integration” (no. 21XTY006) and Beijing Social Science Fund Major Projects “Theoretical and practical research on the deep integration of national fitness and national health in the new era” (no. 20ZDA19).

## Conflict of Interest

The authors declare that the research was conducted in the absence of any commercial or financial relationships that could be construed as a potential conflict of interest.

## Publisher’s Note

All claims expressed in this article are solely those of the authors and do not necessarily represent those of their affiliated organizations, or those of the publisher, the editors and the reviewers. Any product that may be evaluated in this article, or claim that may be made by its manufacturer, is not guaranteed or endorsed by the publisher.

## Footnotes

^1^ www.wjx.cn

## Appendix

Items in the scale of COVID-19 epidemic environment:

1. Due to the COVID-19 epidemic, you feel tired and weak.

2. Due to the COVID-19 epidemic, you are suffering from physical illness caused by sedentary activities such as long-time sitting and lying down.

3. Due to the COVID-19 epidemic, you have to stay at home for a long time and feel listless.

4. Due to the COVID-19 epidemic, you cannot exercise with friends, which makes you feel boring and lack motivation to exercise.

5. Due to the COVID-19 epidemic, there is no place to exercise because of the closure of fitness centres and sports facilities.

6. During the COVID-19 epidemic, you have a poor experience on online exercise activities.

7. Due to the COVID-19 epidemic, sports activities you can participated in are relatively limited, which makes you lose interest in sports.

Note: The items were designed and distributed to participants in Chinese and translated into English later.

## References

[ref1] Ajzen I. (1991). The theory of planned behavior. Organ. Behav. Hum. Decis. Process. 50, 179–211. doi: 10.1016/0749-5978(91)90020-T

[ref2] Ajzen I. Driver B. (1992). Contingent value measurement: On the nature and meaning of willingness to pay. J. Consum. Psychol. 1, 297–316. doi: 10.1016/S1057-7408(08)80057-5

[ref3] Albarracin D. Johnson B. T. Fishbein M. Muellerleile P. A. (2001). Theories of reasoned action and planned behavior as models of condom use: a meta-analysis. Psychol. Bull. 127, 142–161. doi: 10.1037/0033-2909.127.1.142, PMID: 11271752 PMC4780418

[ref4] Ammar A. Brach M. Trabelsi K. Chtourou H. Boukhris O. Masmoudi L. (2020). Effects of COVID-19 home confinement on eating behaviour andm physical activity: results of the ECLB-COVID19 international online survey. Nutrients 12:1583. doi: 10.3390/nu12061583, PMID: 32481594 PMC7352706

[ref5] Ardeńska A. Tomik R. Berber S. Düz B. Çivak B. Çalişkan U.. (2016). A comparison of physical education students’ motivation using polish and Turkish versions of the academic motivation scale. J. Hum. Kinet. 54, 207–218. doi: 10.1515/hukin-2016-0046, PMID: 28031771 PMC5187970

[ref6] Armitage C. J. Conner M. (1999). Distinguishing perception of control from selfefficacy: predicting consumption of a low fat diet using theory of planned behaviour. J. Appl. Psychol. 29, 72–90.

[ref7] Bagherian S. Ghahfarrokhi M. M. Banitalebi E. (2021). Effect of the COVID-19 pandemic on interest in home-based exercise: an application of digital epidemiology. Int. J. Epidemiol. Res. 8, 47–53. doi: 10.34172/ijer.2021.08

[ref8] Bandura A. (1977). Self-efficacy: toward a unifying theory of behavioral change. Psychol. Rev. 84, 191–215. doi: 10.1037/0033-295X.84.2.191, PMID: 847061

[ref9] Bandura A. (1986). Social Foundations of Thought and action: A social Cognitive Theory. Englewood Cliffs, NJ: Prentice Hall.

[ref10] Barkoukis V. Chatzisarantis N. Hagger M. S. (2020). Effects of a school-based intervention on motivation for out-of-school physical activity participation. Res. Q. Exerc. Sport 92, 477–491. doi: 10.1080/02701367.2020.1751029, PMID: 32643561

[ref11] Becker R. K. Maiman B. A. (1975). Siciobehavioral determinants of compliance with health and medical care recommendations. Med. Care 13, 10–24. doi: 10.1097/00005650-197501000-00002, PMID: 1089182

[ref12] Benisovich S. Rossi J. Norman G. Nigg C. (1998). Development of a multidimensional measure of exercise self-efficacy. Ann. Behav. Med. 20.

[ref13] Blue C. L. (2010). The predictive capacity of the theory of reasoned action and the theory of planned behavior in exercise research: an integrated literature review. Res. Nurs. Health 18, 105–121. doi: 10.1002/nur.4770180205 7899566

[ref14] Brodkin P. Weiss M. R. (1990). Developmental differences in motivation for participating in competitive swimming. J. Sport Exerc. Psychol. 12, 248–263. doi: 10.1123/jsep.12.3.248

[ref15] Chang J. Yuan Y. Wang D. (2020). Mental health status and its influencing factors among college students during the epidemic of COVID-19. Nan Fang Yi Ke Da Xue Xue Bao 40, 171–176. doi: 10.12122/j.issn.1673-4254.2020.02.06, PMID: 32376528 PMC7086131

[ref16] Chen S. Wang Y. Rong J. Pan X. Bao J. (2013). Construction of simplified version of exercise motivation scale (MPAM-R) and analysis of its reliability and validity. J. Beijing Univ. Phys. Educ. 36, 66–78.

[ref17] Constandt B. Thibaut E. Bosscher V. D. Scheerder J. Ricour M. Willem A. (2020). Exercising in times of lockdown: an analysis of the impact of covid-19 on levels and patterns of exercise among adults in Belgium. Int. J. Environ. Res. Public Health 17. doi: 10.3390/ijerph17114144, PMID: 32532013 PMC7312512

[ref18] Deci E. L. Ryan R. M. (1985). Intrinsic Motivation and Self-Determination in Human Behaviour. New York: Plenum Press.

[ref19] Diamond R. Waite F. (2020). Physical activity in a pandemic: a new treatment target for psychological therapy. Psychol. Psychother. Theory Res. Pract. 94, 357–364. doi: 10.1111/papt.12294, PMID: 32588499 PMC7361852

[ref20] Duncan L. R. Hall C. R. Wilson P. M. Jenny O. (2010). Exercise motivation: a cross-sectional analysis examining its relationships with frequency, intensity, and duration of exercise. Int. J. Behav. Nutr. Phys. Act. 7, 7–9. doi: 10.1186/1479-5868-7-7 20181017 PMC2835648

[ref21] Dwyer M. J. Pasini M. De Dominicis S. Righi E. (2020). Physical activity: benefits and challenges during the COVID-19 epidemic. Scand. J. Med. Sci. Sports 30, 1291–1294. doi: 10.1111/sms.13710, PMID: 32542719 PMC7323175

[ref22] Dzewaltowski D. A. (1989). Towards a model of exercise motivation. J. Sport Exerc. Psychol. 11, 251–269. doi: 10.1123/jsep.11.3.251

[ref23] Farah B. Q. Prado W. L. Malik N. Lofrano-Prado M. C. Melo P. H. Botero J. P.. (2021). Barriers to physical activity during the COVID-19 pandemic in adults: a cross-sectional study. Sport Sci. Health 17, 441–447. doi: 10.1007/s11332-020-00724-5, PMID: 33815618 PMC7998080

[ref24] Godinic D. Obrenovic B. Khudaykulov A. (2020). Effects of economic uncertainty on mental health in the covid-19 pandemic context: social identity disturbance, job uncertainty and psychological well-being model. Int. J. Innov. Econ. Dev. 6, 61–74. doi: 10.18775/ijied.1849-7551-7020.2015.61.2005

[ref25] Guberina T. Wang A. M. (2021). Entrepreneurial leadership impact on job security and psychological well-being during the COVID-19 pandemic: a conceptual review. Int. J. Innov. Econ. Dev. 6, 7–18. doi: 10.18775/ijied.1849-7551-7020.2015.66.2001

[ref26] Hagger M. S. Chatzisarantis N. L. D. Biddle S. J. H. (2002). The influence of autonomous and controlling motives on physical activity intentions within the theory of planned behaviour. Br. J. Health Psychol. 7, 283–297. doi: 10.1348/135910702760213689, PMID: 12614501

[ref27] Hooper D. Coughlan J. Mullen M. R. (2008). Structural equation modelling: guidelines for determining model fit. Electron. J. Bus. Res. Methods 6, 141–146.

[ref28] Hu D. Zong B. Wan B. (2020). Study on the behavior and promotion of undergraduate students' physical exercise at home during the epidemic of COVID-19. J. Wuhan Instit. Phys. Educ. 54, 80–86.

[ref29] Karimy M. Zareban I. Araban M. Montazeri A. (2015). An extended theory of planned behavior (TPB) used to predict smoking behavior among a sample of Iranian medical students. Int. J. High Risk Behav. Addict. 4:e24715. doi: 10.5812/ijhrba.24715, PMID: 26495261 PMC4609501

[ref30] Kaur H. Singh T. Arya Y. K. Mittal S. (2020). Physical fitness and exercise during the covid-19 pandemic: a qualitative enquiry. Front. Psychol. 11. doi: 10.3389/fpsyg.2020.590172, PMID: 33250827 PMC7673425

[ref31] Khudaykulov A. Changjun Z. Obrenovic B. Godinic D. Alsharif H. Z. H. Jakhongirov I. (2022). The fear of covid-19 and job insecurity impact on depression and anxiety: an empirical study in China in the covid-19 pandemic aftermath. Curr. Psychol. 1–14. doi: 10.1007/s12144-022-02883-9 PMC8906526 35287294

[ref32] Klompstra L. Jaarsma T. Strömberg A. (2018). Self-efficacy mediates the relationship between motivation and physical activity in patients with heart failure. J. Cardiovasc. Nurs. 33, 211–216. doi: 10.1097/JCN.0000000000000456, PMID: 29189427 PMC5908261

[ref33] Kwan B. M. Bryan A. D. (2010). Affective response to exercise as a component of exercise motivation: attitudes, norms, self-efficacy, and temporal stability of intentions. Psychol. Sport Exerc. 11, 71–79. doi: 10.1016/j.psychsport.2009.05.010, PMID: 20161385 PMC2782828

[ref34] Lavie C. J. Ozemek C. Carbone S. Katzmarzyk P. T. Blair S. N. (2019). Sedentary behavior, exercise, and cardiovascular health. Circ. Res. 124, 799–815. doi: 10.1161/CIRCRESAHA.118.312669 30817262

[ref35] Lee J. Jeong H. J. Kim S. (2021). Stress, anxiety, and depression among undergraduate students during the COVID-19 pandemic and their use of mental health services. Innov. High. Educ. 46, 519–538. doi: 10.1007/s10755-021-09552-y, PMID: 33907351 PMC8062254

[ref36] Li Y. (2010). The Relationship between Exercise Intention and Behavior: The Role of Planning, Self-Efficacy, and social Support. Beijing: Beijing University of Physical Education.

[ref37] Li N. (2018). The development and test of undergraduate students' physical exercise behavior questionnaire. Guide Sci. Educ. 18, 214–215.

[ref38] Liao C. Chen J. L. Yen D. C. (2007). Theory of planning behavior (tpb) and customer satisfaction in the continued use of e-service: an integrated model. Comput. Hum. Behav. 23, 2804–2822. doi: 10.1016/j.chb.2006.05.006

[ref39] Lim K. C. (2009). University students’ attitude, self-efficacy and motivation regarding leisure time physical participation. J. Educat. Educ. 24, 1–15.

[ref40] Lim H. J. Lim H. S. Lee M. S. (2005). Relationship between self-efficacy and exercise duration in patients with ankylosing spondylitis. Clin. Rheumatol. 24, 442–443. doi: 10.1007/s10067-004-0974-8, PMID: 15338448

[ref41] Liu S. Liu Y. Liu Y. (2020). Somatic symptoms and concern regarding COVID-19 among Chinese college and primary school students: a cross-sectional survey. Psychiatry Res. 289, 113070–113075. doi: 10.1016/j.psychres.2020.113070, PMID: 32422501 PMC7227526

[ref42] Maslow A. H. (1943). A theory of human motivation. Psychol. Rev. 50, 370–396. doi: 10.1037/h0054346

[ref43] Maugeri G. Castrogiovanni P. Battaglia G. Pippi R. D'Agata V. Palma A.. (2020). The impact of physical activity on psychological health during Covid-19 epidemic in Italy. Heliyon 6:e04315. doi: 10.1016/j.heliyon.2020.e04315 32613133 PMC7311901

[ref44] McAuley E. Blissmer B. Katula J. Duncan T. E. Mihalko S. L. (2000). Physical activity, self-esteem, and self-efficacy relationships in older adults: a randomized controlled trial. Ann. Behav. Med. 22, 131–139. doi: 10.1007/BF02895777 10962706

[ref45] McAuley E. Jerome G. J. Elavsky S. Marquez D. X. Ramsey S. N. (2003). Pedicting long-term maintenance of physical activity in older adults. Prev. Med. 37, 110–118. doi: 10.1016/S0091-7435(03)00089-6, PMID: 12855210

[ref46] Mutz M. Gerke M. (2020). Sport and exercise in times of self-quarantine: how Germans changed their behaviour at the beginning of the Covid-19 pandemic. Int. Rev. Sociol. Sport 56, 305–316. doi: 10.1177/1012690220934335

[ref47] National Center for Complementary and Integrative Health (2020). Yoga: what you need to know. Available at: https://nccih.nih.gov/health/yoga/introduction.htm (Accessed March 9, 2022).

[ref48] Rodríguez M. Á. Crespo I. Olmedillas H. (2020). Exercising in times of COVID-19: what do experts recommend doing within four walls? Revista Espanola De Cardiologia 73, 527–529. doi: 10.1016/j.rec.2020.04.001, PMID: 32414660 PMC7142674

[ref49] Rogers R. W. (1983). Cognitive and physiological processes in fear appeals and attitude change: a revised theory of protection motivation. Soc. Psychophys. 19, 469–479.

[ref50] Ryan R. M. Frederick C. M. Lepes D. Rubio N. Sheldon K. M. (1997). Intrinsic motivation and exercise adherence. Int. J. Sport Psychol. 28, 335–354.

[ref51] Saddik B. Hussein A. Sharif-Askari F. S. Kheder W. Temsah M. H. Koutaich R. A.. (2020). Increased levels of anxiety among medical and non-medical university students during the COVID-19 pandemic in the United Arab Emirates. Risk Manag. Healthc. Policy 13, 2395–2406. doi: 10.2147/RMHP.S273333, PMID: 33177898 PMC7652570

[ref52] Schinke R. Papaioannou A. Maher C. Parham W. D. Larsen C. H. Gordin R.. (2020). Sport psychology services to professional athletes: working through COVID-19. Int. J. Sport Exerc. Psychol. 18, 409–413. doi: 10.1080/1612197X.2020.1766182

[ref53] Schnitzer M. Schöttl S. E. Kopp M. Barth M. (2020). COVID-19 stay-at-home order in Tyrol, Austria: sports and exercise behaviour in change? Public Health 185, 218–220. doi: 10.1016/j.puhe.2020.06.042, PMID: 32659514 PMC7318923

[ref54] Schwendinger F. Pocecco E. (2020). Counteracting physical inactivity during the covid-19 pandemic: evidence-based recommendations for home-based exercise. Int. J. Environ. Res. Public Health 17. doi: 10.3390/ijerph17113909, PMID: 32492778 PMC7311977

[ref55] Spence J. C. Lee R. E. (2003). Toward a comprehensive model of physical activity. Psychol. Sport Exerc. 4, 7–24. doi: 10.1016/S1469-0292(02)00014-6

[ref56] Standage M. Gillison F. B. Ntoumanis N. Treasure D. C. (2012). Predicting students' physical activity and health-related well-being: a prospective cross-domain investigation of motivation across school physical education and exercise settings. J. Sport Exerc. Psychol. 34, 37–60. doi: 10.1123/jsep.34.1.37, PMID: 22356882

[ref57] Tate D. F. Lyons E. J. Valle C. G. (2015). High-tech tools for exercise motivation: use and role of technologies such as the internet, mobile applications, social media, and video games. Diabetes Spectr. 28, 45–54. doi: 10.2337/diaspect.28.1.45, PMID: 25717278 PMC4334081

[ref58] Terry D. J. Hogg M. A. White K. M. (1999). The theory of planned behaviour: self-identity, social identity and group norms. Br. J. Soc. Psychol. 38, 225–244. doi: 10.1348/014466699164149, PMID: 10520477

[ref59] Varea V. González-Calvo G. (2020). Touchless classes and absent bodies: teaching physical education in times of Covid-19. Sport Educ. Soc. 26, 831–845. doi: 10.1080/13573322.2020.1791814

[ref60] Woods J. Hutchinson N. T. Powers S. K. Roberts W. O. Gomez-Cabrera M. C. Radak Z.. (2020). The COVID-19 pandemic and physical activity. Sports Med. Health Sci. 2, 55–64. doi: 10.1016/j.smhs.2020.05.006, PMID: 34189484 PMC7261095

[ref61] WTTC (2020). Latest research from WTTC shows a 50% increase in jobs at risk in Travel and Tourism. Available at: https://wttc.org/News-Article/Latest-research-from-WTTC-shows-a-50-percentage-increase-in-jobs-at-risk-in-Travel-and-Tourism (Accessed January 14, 2021).

[ref62] Yarımkaya E. Esentürk O. K. (2020). Promoting physical activity for children with autism spectrum disorders during coronavirus outbreak: benefits, strategies, and examples. Int. J. Dev. Disabil. 1–6. doi: 10.1080/20473869.2020.1756115 PMC9351550 35937165

[ref63] Yeo T. J. (2020). Sport and exercise during and beyond the COVID-19 epidemic. Eur. J. Prev. Cardiol. 27, 1239–1241. doi: 10.1177/2047487320933260, PMID: 32539555 PMC7717326

[ref64] Yordy G. A. Lent R. W. (1993). Predicting aerobic exercise participation: social cognitive, reasoned action and planned behaviour models. J. Sport Exerc. Psychol. 15, 363–374. doi: 10.1123/jsep.15.4.363

[ref65] Zhang S. X. Wang Y. Rauch A. Wei F. (2020b). Unprecedented disruption of lives and work: health, distress and life satisfaction of working adults in China one month into the COVID-19 outbreak. Psychiatry Res. 288, 112958–111158. doi: 10.1016/j.psychres.2020.112958 32283450 PMC7146665

[ref66] Zhang J. Zhong C. Zhan X. (2020a). A study on the physical exercise behavior and its influencing factors of undergraduate students during the epidemic of COVID-19—a case study of Jiangxi Normal University. Youth Sports 4, 23–63.

